# Intrapleural Adenoviral-mediated Endothelial Cell Protein C Receptor Gene Transfer Suppresses the Progression of Malignant Pleural Mesothelioma in a Mouse Model

**DOI:** 10.1038/srep36829

**Published:** 2016-11-11

**Authors:** Shiva Keshava, L. Vijaya Mohan Rao, Usha R. Pendurthi

**Affiliations:** 1 Department of Cellular and Molecular Biology, The University of Texas Health Science Center at Tyler, Texas, USA

## Abstract

Malignant pleural mesothelioma (MPM) is an aggressive thoracic cancer with a high mortality rate as it responds poorly to standard therapeutic interventions. Our recent studies showed that expression of endothelial cell protein C receptor (EPCR) in MPM cells suppresses tumorigenicity. The present study was aimed to investigate the mechanism by which EPCR suppresses MPM tumor growth and evaluate whether EPCR gene therapy could suppress the progression of MPM in a mouse model of MPM. Measurement of cytokines from the pleural lavage showed that mice implanted with MPM cells expressing EPCR had elevated levels of IFNγ and TNFα compared to mice implanted with MPM cells lacking EPCR. *In vitro* studies demonstrated that EPCR expression renders MPM cells highly susceptible to IFNγ + TNFα-induced apoptosis. Intrapleural injection of Ad.EPCR into mice with an established MPM originating from MPM cells lacking EPCR reduced the progression of tumor growth. Ad.EPCR treatment elicited recruitment of macrophages and NK cells into the tumor microenvironment and increased IFNγ and TNFα levels in the pleural space. Ad.EPCR treatment resulted in a marked increase in tumor cell apoptosis. In summary, our data show that EPCR expression in MPM cells promotes tumor cell apoptosis, and intrapleural EPCR gene therapy suppresses MPM progression.

Endothelial cell protein C receptor (EPCR) was first identified and isolated as a cellular receptor for protein C on endothelial cells^1^. EPCR plays a crucial role in the protein C anticoagulant pathway by promoting protein C activation^2^. EPCR also serves as the cellular receptor for activated protein C (APC) and supports APC-mediated vascular protective signaling via activation of protease-activated receptors. (PARs)^3^,^4^. Although originally identified as an endothelial cell receptor, EPCR has since been detected in a variety of cell types^5^, including hematopoietic, epithelial progenitor cells, and cancer cells^6^,^7^,^8^,^9^. Recent studies discovered novel ligands for EPCR^4^, such as *Plasmodium falciparum* erythrocyte membrane protein 1^10^, and a specific variant of the T-cell receptor^11^. These observations have opened unsuspected new roles for EPCR beyond hemostasis^4^.

EPCR-mediated cell signaling, in general, was shown to contribute to cell survival and anti-apoptotic pathways^3^,^4^,^12^. EPCR-APC-induced cell signaling was shown to inhibit apoptosis in endothelial cells, cancer cells, and other cell types^13^,^14^,^15^,^16^,^17^. The EPCR-APC axis promoted the survival of lung adenocarcinoma cells by preventing their apoptosis^18^. EPCR expressing breast cancer stem cells were shown to have increased tumor cell-initiating activity compared to cells lacking EPCR^19^. EPCR overexpression in breast cancer cells increased the tumor growth potential at an initial stage^20^. Surprisingly, our recent studies revealed that EPCR could function as a negative regulator of cancer progression in malignant pleural mesothelioma (MPM)^21^. These studies showed that transduction of EPCR gene expression in aggressive REN MPM cells that express oncogenic tissue factor (TF) but lack EPCR markedly attenuated the tumorigenicity of REN MPM cells^21^. Confirming the tumor suppressive effect of EPCR in MPM, the knock-down of EPCR in non-aggressive TF expressing MPM cells that constitutively express EPCR increased the tumorigenicity of the non-aggressive MPM cells^21^. This study also revealed that EPCR in MPM cells promotes tumor cell apoptosis *in vivo.*

Malignant pleural mesothelioma is a highly aggressive form of thoracic cancer and an exceedingly difficult disease to treat with currently available therapies^22^,^23^. Exposure to airborne asbestos is the primary cause of the development of MPM^24^. However, exposure to other mineral fibers such as erionite, simian virus 40, and radiation are also thought to be causative agents for the development of MPM^25^,^26^,^27^,^28^. Recent studies suggest that carbon nanotubes, which are increasingly used in manufacturing, might share the carcinogenic mechanism postulated for asbestos and could induce mesothelioma^29^. Although MPM is a much rarer disease than other cancers, it still kills about 2,500 people/year in the US alone^30^. Furthermore, the incidence of MPM is expected to increase, particularly in 3^rd^ world countries, due to the widespread exposure to asbestos in recent past decades and a long latency period associated with MPM development^28^. If left untreated, the median survival time of MPM patients is about 12 months^28^. Although a variety of treatment modalities, such as surgical treatment, chemotherapy, radiotherapy, and immunotherapy, has been developed to treat MPM, the efficacy of these treatments is very limited; they improved the quality of life and prolonged the survival of MPM patients only modestly^28^,^31^. It was thought that MPM might be a good target for gene therapy since no effective therapies exist currently. Furthermore, the disease remains relatively localized until late in its course, and the tumor can be accessed relatively easily through the chest wall^32^. A few phase I trials with cytokine gene therapy have been carried out in MPM patients and these trials have shown feasibility and safety but only limited efficacy^32^,^33^,^34^,^35^,^36^.

Since our recent studies clearly showed that transduction of EPCR expression in highly aggressive MPM cells that lack EPCR promoted tumor cell apoptosis and suppressed the tumor growth^21^, the present study was undertaken to investigate whether intrapleural EPCR gene transfer will have therapeutic value in halting the progression of MPM. The data presented herein provide evidence to a proof-of-principle that intrapleural EPCR gene therapy has the potential to slow the progression of MPM.

## Results

### Increased levels of TNFα and IFNγ in pleural lavage of mice implanted with REN(+EPCR) MPM xenograft

**Figure 1:**
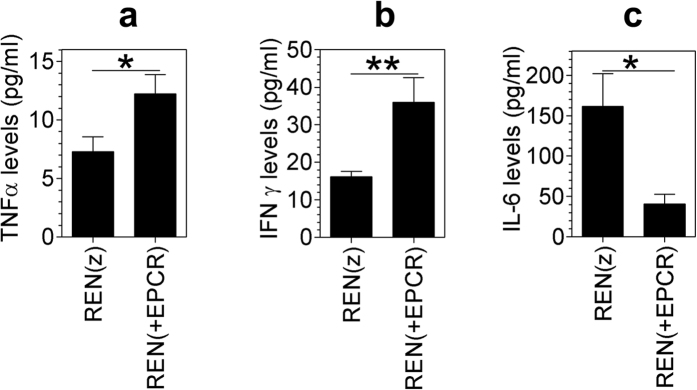
IFNγ, TNFα and IL-6 levels in the pleural lavage of nude mice implanted with REN and REN(+EPCR) MPM cells in the pleural cavity. REN(z) or REN(+EPCR) MPM cells (1 × 10^6^ cells) were injected into the thoracic cavity of nude mice (BALB/c, NU/J). At the end of 30 days following tumor cell implantation, mice were euthanized, tumor tissues and pleural lavages were collected. Cytokine levels in the pleural lavage were quantified by ELISA. (**a**) TNFα; (**b**) IFNγ; (**c**) IL-6. *p < 0.05; **p < 0.01 (n = 11 to 15 mice/group).

Our recent study showed that EPCR functions as a crucial negative regulator of MPM progression in a mouse model^21^. This study revealed that tumors originating from MPM cells expressing EPCR were more apoptotic compared to tumors originating from MPM cells lacking EPCR. Since IFNγ alone or in combination with TNFα was shown to exhibit anti-tumor activity including in MPM patients^37^,^38^, we investigated whether EPCR expression in MPM cells leads to an increase in levels of these cytokines in the thoracic cavity. To investigate this, we have measured levels of TNFα and IFNγ in the pleural lavage of mice implanted with REN(z) (control vector transfected cells) or REN(+EPCR) MPM cells orthotopically. Evaluation of thoracic fluids obtained from nude mice implanted with the above MPM cell types showed a significant increase (a 2 to 3-fold increase) in the levels of mTNFα and mIFNγ in mice implanted with REN (+EPCR) cells compared to mice implanted with REN(z) cells (Fig. 1a,b). In contrast to the increased TNFα and IFNγ levels, IL-6 levels were reduced in the pleural lavage of mice implanted with REN(+EPCR) MPM cells compared to mice implanted with REN(z) cells (Fig. 1c).

### EPCR renders MPM cells susceptible to TNFα + IFNγ-induced apoptosis

**Figure 2:**
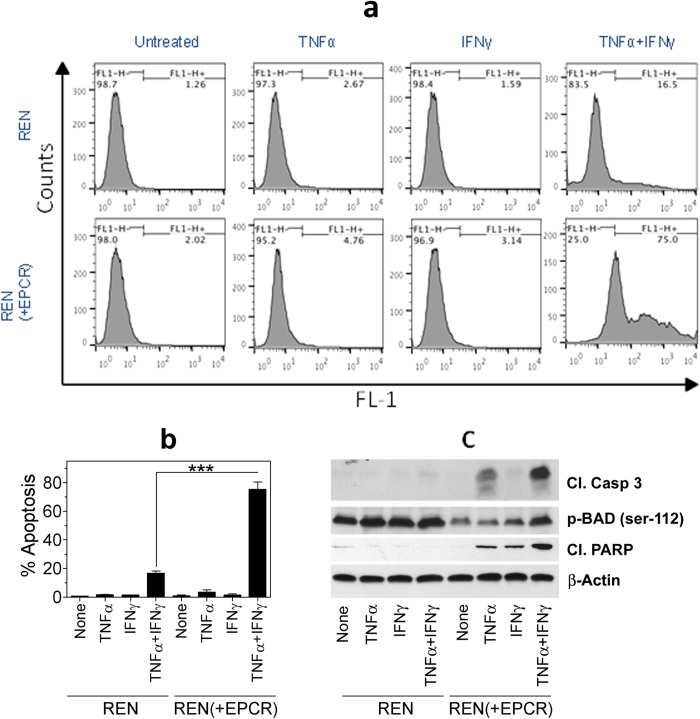
Transduction of EPCR expression in MPM cells promotes apoptosis. Monolayers of REN MPM cells that lack EPCR expression were stably transfected with empty vector or EPCR expression vector. REN and REN(+EPCR) cells were treated with TNFα (10 ng/ml), IFNγ (100 U/ml) or TNFα + IFNγ (10 ng and 100 U/ml) or none (untreated/control) in 10% v/v serum-containing medium. After 72 h, cells were harvested and processed for TUNEL assay. The samples were analyzed using BD FACS Calibur. (**a**) Flow cytometry data from a typical experiment. (**b**) Mean data from three independent experiments. ***p < 0.001. (**c**) REN and REN(+EPCR) cells were treated with TNFα (10 ng/ml), IFNγ (100 U/ml) or TNFα + IFNγ (10 ng and 100 U/ml) for 24 h and cell extracts were subjected to western blot analysis and probed for apoptotic markers, cleaved caspase 3 (cl. Casp 3), cleaved poly (ADP-ribose) polymerase (cl. PARP), and p-BAD.

**Figure 3:**
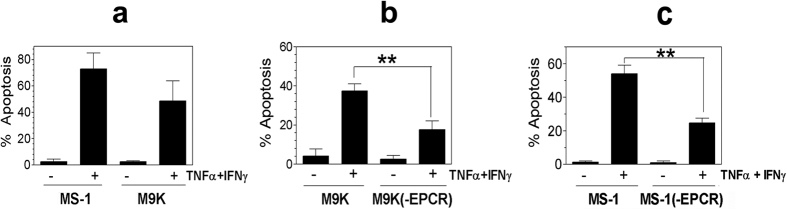
MPM cells constitutively expressing EPCR are susceptible to IFNγ/TNFα-induced apoptosis and the knock-down of EPCR suppresses apoptosis. (**a**) MS-1 and M9K MPM cells, which constitutively express EPCR, were treated with a combination of TNFα 10 ng/ml) + IFNγ (100 U/ml) for 72 h in the serum-containing medium. At the end of 72 h, the cells were harvested and processed for measuring apoptosis by flow cytometry using TUNEL staining. (**b**) M9K MPM cells and M9K MPM cells that were transfected with shEPCR to knock-down the EPCR expression (M9K(-EPCR)) were treated with TNFα + IFNγ as described in Panel **a** and the extent of apoptosis was measured. (**c**) MS-1 cells expressing tissue factor (MS-1) and MS-1 cells transfected with shEPCR to knock-down the EPCR expression (MS-1(-EPCR)) were treated with TNFα + IFNγ as described in Panel **a** and the extent of apoptosis was measured. **p < 0.01.

To investigate the effect of EPCR expression on MPM cells on tumor cell apoptosis, REN and REN(+EPCR) cells were treated with a control vehicle, TNFα, IFNγ or TNFα + IFNγ for 72 h and tumor cell apoptosis was evaluated by TUNEL assay. As shown in Fig. 2a,b, treatment of REN or REN(+EPCR) cells with either TNFα or IFNγ alone had no significant effect on tumor cell apoptosis compared to control vehicle treated cells. In all cases, tumor cell apoptosis was minimal (<2%). A combination of TNFα + IFNγ treatment increased REN MPM cell apoptosis to about 15 to 20%. More importantly, transduction of EPCR expression in REN MPM cells markedly increased the TNFα + IFNγ-induced cell death in REN cells. TNFα + IFNγ treatment killed approximately 80% of REN(+EPCR) cells. Evaluation of apoptotic markers in REN and (REN + EPCR) cells by western blot analysis showed increased levels of cleaved caspase 3 and cleaved PARP, and reduced levels of p-BAD in REN(+EPCR) cells treated with TNFα + IFNγ compared to REN cells treated TNFα + IFNγ (Fig. 2c). These data further confirm that EPCR expressing MPM cells were more susceptible to TNFα + IFNγ-induced apoptosis. It is important to note here that the effect of EPCR on promoting TNFα + IFNγ-induced cell death is not a clonal artifact. The data were reproduced with multiple clones and a stable transfectant that were not subjected to clonal selection. Furthermore, the results were reproduced in independent transfections. Consistent with the concept that EPCR expression in MPM cells promotes apoptosis, MS-1 and M9K MPM cells that constitutively express EPCR were highly susceptible to TNFα + IFNγ-induced cell death (Fig. 3a). Confirming the role of EPCR in rendering MPM cells susceptible to TNFα + IFNγ-induced cell death, the knock-down of EPCR in both M9K and MS-1 MPM cells significantly reduced the extent of cytokine-induced cell death (Fig. 3b,c).

### Transduction of EPCR expression to established REN MPM xenograft slows the progression of MPM

**Figure 4:**
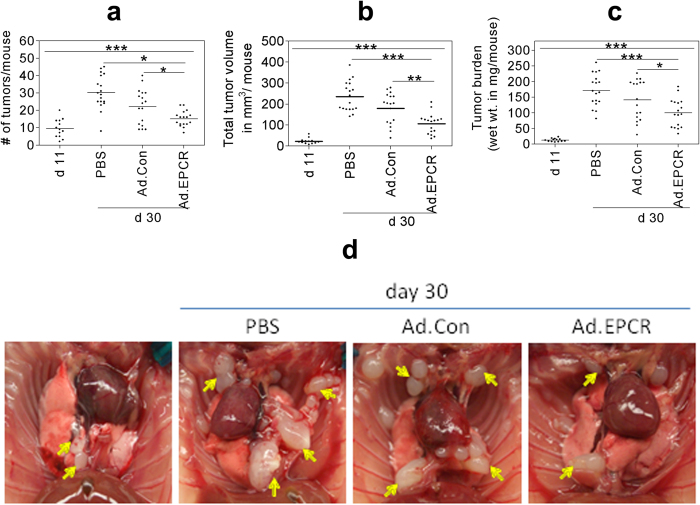
Ad.EPCR treatment curtails the progression of MPM in the mouse model. Nude mice (BALB/c, NU/J) were implanted with 1 × 10^6^ REN MPM cells in the thoracic cavity. On the 11th day (d 11) following the tumor cell implantation, a group of mice was killed to monitor the establishment of MPM tumors in the thoracic cavity. Then the remaining mice were divided randomly into three groups, and each group of mice was injected intrapleurally with either sterile PBS (100 μl), the control adenovirus (Ad.Con), or EPCR adenovirus (Ad.EPCR) (2 × 10^9^ pfu in 100 μl PBS, once every three days). At the end of 30 days (d 30), mice were euthanized, and tumor statistics were recorded. **(a)** tumor count; **(b)** tumor volume; **(c)** tumor burden; **(d)** a representative photograph showing differences in tumor growth in mice treated with PBS, control adenovirus or EPCR adenovirus following REN MPM cell implantation for 10 days. Arrows indicate tumors. A total of three independent experiments were conducted with a total of 16–18 mice/group. *p < 0.05; **p < 0.01; ***p < 0.001.

**Figure 5:**
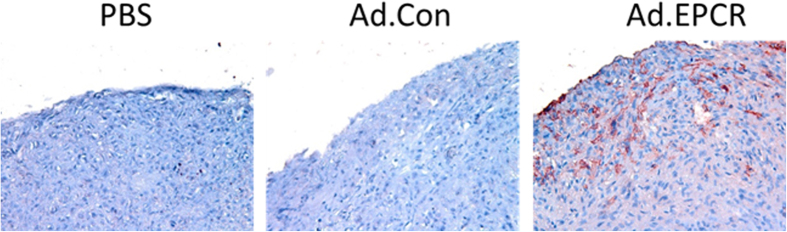
EPCR expression in MPM tumors following the administration of Ad.EPCR. NU/J mice were implanted with REN MPM cells and then treated with a control adenovirus or the adenovirus encoding EPCR as described in Fig. 4. At the end of the 30-day experimental period, tumors were excised and then processed for tissue sectioning and immunostaining with EPCR antibody. Red staining in the right panel of the micrograph indicates EPCR expression in tumor cells.

As the above data in cell model systems clearly showed that EPCR expression in MPM cells promotes apoptosis, we next investigated whether transduction of EPCR expression to aggressively growing REN tumors would reduce the tumor growth *in vivo*. To test this possibility, we first generated adenoviral vectors expressing either human EPCR or a control protein (GFP), prepared high-titer viral stocks, and purified the virus. Nude mice (BALB/c, NU/J, Stock number 002019, Jackson Laboratories, Maine) were implanted with 1 × 10^6^ aggressive REN MPM cells that lack EPCR in a growth factor-reduced Matrigel in the intrapleural space. On the 11^th^ day following the tumor cell implantation, a group of mice was killed to monitor the establishment of the MPM tumors in the thoracic cavity. The remaining mice were divided randomly into three groups, and each group of mice was injected intrapleurally with either sterile PBS (100 μl), Ad.Control, or Ad.EPCR (2 × 10^9^ particles in 100 μl PBS). The intrapleural administration of PBS, Ad.Control or Ad.EPCR was repeated in three-day intervals. At the end of 30 days, mice were euthanized, and thoracic cavities were opened; tumor statistics were recorded (Fig. 4a) and the exposed thoracic cavity was photographed (Fig. 4b). A total of three independent experiments were conducted with a total of 16–18 mice/group. Analysis of the resultant data revealed that mice receiving EPCR adenovirus showed a significant decrease in tumor number, tumor volume, and tumor burden compared to the group of mice receiving PBS (p < 0.001 in both t-test and ANOVA) or the control adenovirus (p < 0.01 for tumor volume in both t-test and ANOVA; p < 0.05 for tumor number and burden in t-test) (Fig. 4a). Somewhat less pronounced differences in tumor statistics between Ad.EPCR virus and Ad.Control injected mice reflect a slight protective effect of the control adenovirus in comparison to PBS control. Immunohistochemical analysis of tumor tissue sections derived from tumors excised at the end of the 30-day experimental period showed EPCR expression in tumor cells in mice injected with EPCR adenovirus but not in the control adenovirus or PBS. EPCR expression is more abundant in tumor cells present in the periphery (several cell thickness) of tumor tissue whereas it is sparse in the tumor core (Fig. 5).

**Figure 6:**
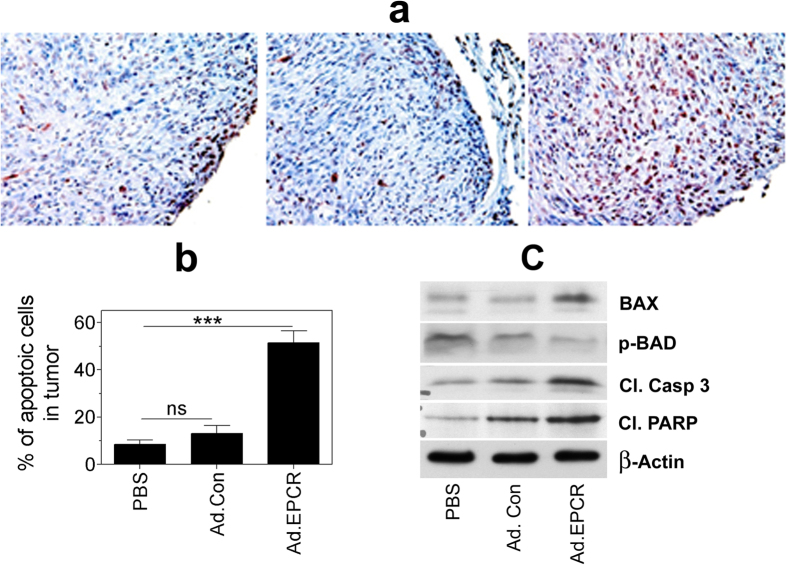
Transduction of EPCR expression in REN MPM tumors lacking EPCR induces tumor cell apoptosis. NU/J nude mice were implanted with REN MPM cells and 10 days following the tumor cell implantation, mice were treated with PBS, control adenovirus or adenovirus encoding EPCR for 20 days as described in Fig. 4. At the end of the 30-day experimental period, tumors were removed and processed for tissue sectioning or preparing tissue extracts. Tissue sections were immunostained for TUNEL staining (panel **a**), and the percentage of apoptotic tumor cells was determined by counting tumor cells stained positive for TUNEL staining and the total number of tumor cells in that field (panel **b**). Tumor tissue extracts were subjected to western blot analysis and probed for apoptotic markers, Bax, p-BAD, cleaved caspase 3 (cl. Casp 3), and cleaved poly (ADP-ribose) polymerase (cl. PARP) (panel **c**). Panel (**a**,**c**) depict representative images. Cells stained with dark red color in panel **a** indicate apoptotic cells. Data shown in panel **b** were obtained by counting the number of apoptotic cells and the total number of tumor cells present in 20 to 25 randomly selected fields from 5 or more tumors derived from different mice. ***p < 0.001.

TUNEL staining of tumor tissue sections to assess tumor cell apoptosis revealed that treatment of mice with EPCR adenovirus markedly increased tumor cell apoptosis (Fig. 6a,b). In animals treated with saline or Ad.Control, about 10% of tumor cells were apoptotic whereas in Ad.EPCR treated animals, approximately 50 to 60% of tumor cells were apoptotic (Fig. 6b). Analysis of tumor tissue extracts by western blot analysis showed increased levels of proapoptotic protein, BAX, decreased p-BAD, and increased levels of cleaved caspase 3 and PARP in tumor tissues obtained from mice treated with Ad.EPCR compared to mice treated with PBS or Ad.Con (Fig. 6c). These data suggest a greater extent of apoptosis in tumors of mice that were treated with Ad.EPCR compared to mice treated with a control vehicle or control vector. These data further strengthen our observation that EPCR adenovirus treatment promotes tumor apoptosis in MPM.

### Ad.EPCR treatment enhances the recruitment of macrophages and natural killer cells into tumor microenvironment and alters elaboration of inflammatory cytokines/chemokines in the pleural space

**Figure 7:**
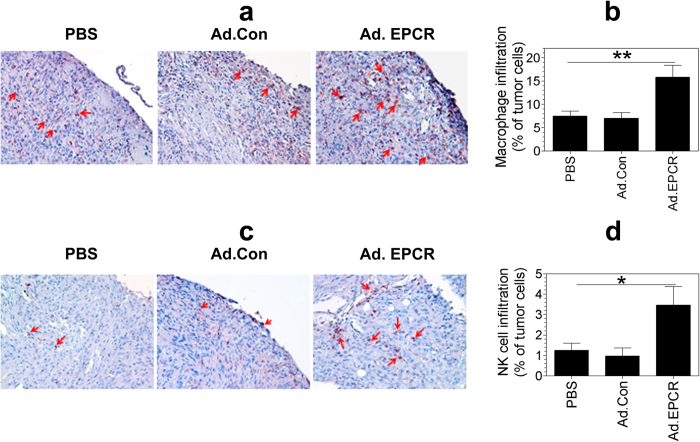
Transduction of EPCR expression in MPM recruits macrophages and NK cells into tumor environment. Nu/J nude mice were implanted with REN MPM cells and 10 days following the tumor cell implantation, mice were treated with PBS, control adenovirus or adenovirus encoding EPCR for 20 days as described in Fig. 4. At the end of the 30-day experimental period, tumors were removed and processed for tissue sectioning. Tissue sections were stained for the expression of mouse macrophage marker F4/80 **(a)** or NK cell marker NK1.1 **(c).** The percentage of macrophages and NK cells in tumors was determined by counting the number of cells stained positive for either F4/80 or NK1.1 in a field and the number of tumor cells present in that field. Images shown in panels (**a**,**c)** are representative images. Cells stained with red color indicate macrophages (**a**) and NK cells (**c**; also identified by arrows). Data shown in panels (**b**,**d**) were obtained by counting the number of positively stained cells and the total number of tumor cells in 20 to 25 randomly selected fields from 5 or more tumors derived from different mice. *p < 0.05; **p < 0.01.

Staining of tissue sections of tumors with mouse macrophage marker F4/80 showed the presence of few infiltrating macrophages (~5 to 8%) in the tumor microenvironment of tumors derived from mice treated with either saline or control adenovirus (Fig. 7a). Analysis of tissue sections of tumors derived from mice treated with EPCR adenovirus showed a significant increase in the number of macrophages (greater than 15%) recruited into tumors (Fig. 7b). Similarly, Ad. EPCR treatment also increased the recruitment of NK cells into tumors (Fig. 7c), from ~1% seen in saline or Ad.Control treated animals to ~4% in Ad.EPCR treated animals (Fig. 7d).

**Figure 8:**
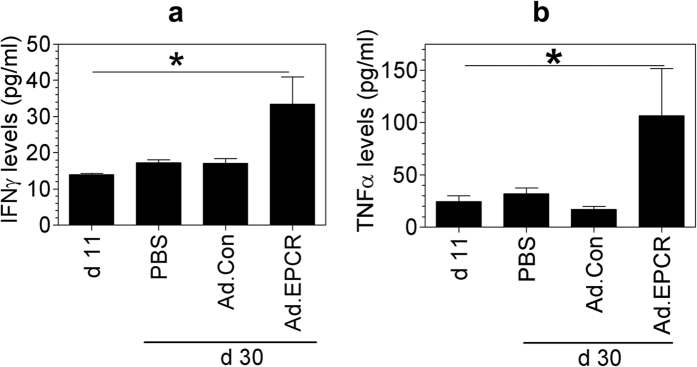
IFNγ and TNFα levels in the pleural lavage of nude mice implanted with REN and treated with PBS, control adenovirus or EPCR adenovirus. REN MPM cells (1 × 10^6^ cells) were injected into the thoracic cavity of nude mice. After 10 days, mice were treated with PBS, control adenovirus or EPCR adenovirus as described in Fig. 4. At the end of 30 days following tumor cell implantation, mice were euthanized, and pleural lavages were collected. Cytokine levels in the pleural lavage were quantified using ELISA (**a**) TNFα; (**b**) IFNγ (n = 13 to 16 mice/group). *p < 0.05 in one-way ANOVA. Values in Ad.EPCR treated animals also differ in a statistically significant manner from PBS or Ad.Con treated animals.

Measurement of TNFα and IFNγ levels in the pleural lavage showed a significant increase in TNFα and IFNγ levels in mice treated with EPCR adenovirus but not in mice treated with saline or control adenovirus (Fig. 8a,b). Although as shown in Fig. 4c, the tumor burden was markedly increased at day 30 compared to day 11, there were no significant differences in TNFα and IFNγ levels in the pleural lavage obtained from mice at day 11 (before the initiation of the treatment) or day 30 that were treated with saline or control adenovirus (Fig. 8a,b). In addition to measuring TNFα and IFNγ levels, we also measured using the multiplex assay the levels of a panel of inflammatory cytokines and chemokines in the pleural lavage of mice treated with saline, control adenovirus, and EPCR adenovirus and compared them to the levels measured at day 11, i.e., beginning of the treatment. The levels of IL-3, IL-4, IL-5, IL-6, IL-12, IL-13, G-CSF, MIP-ß and RANTES levels were increased by 2 to 4-fold at day 30 (PBS treatment) in comparison to the levels measured at day 11, at the beginning of the treatment (Table 1). Treatment of mice with EPCR adenovirus and not the control adenovirus significantly decreased the levels of IL-3, IL-4, IL-6, IL-12 and RANTES. In the case of G-CSF and MCP-1, both control adenovirus and EPCR adenovirus treatment significantly reduced their levels compared to PBS treatment.

## Discussion

Our recent studies, using MPM cell types derived from human patients in a mouse MPM xenograft model, showed that EPCR expression in MPM cells attenuates TF-driven MPM tumor growth^21^. This observation raised a possibility that EPCR gene therapy may curtail MPM tumor growth and progression. The data presented here provide proof-of-principle evidence to this concept. The data show that intrapleural EPCR gene transfer to aggressively growing MPM using adenoviral vector reduced MPM tumor growth in a murine model of MPM. The data show that EPCR gene transfer to MPM cells induces tumor apoptosis, both *in vitro* and *in vivo.*

The primary function of EPCR is the regulation of the protein C anticoagulant pathway^39^. EPCR is constitutively expressed on the endothelium^40^. The ability of primary pleural mesothelial cells to support protein C binding and thrombin-dependent activation of protein C^41^ indicates that primary pleural mesothelial cells constitutively express EPCR. Many cancer cell types and cancer tissues were also shown to express EPCR^42^,^43^,^44^,^45^,^46^. However, the role of EPCR in cancer pathogenesis is unclear. Recent studies provide convincing evidence that EPCR functions go beyond its role in coagulation as it was shown to bind multiple receptors and support APC-induced cell signaling^4^. Since EPCR-mediated cell signaling typically activates cell survival and anti-apoptotic pathways^3^,^4^, it is generally believed that EPCR expression in tumor cells promotes tumor growth and metastasis^47^,^48^. Supporting this notion, EPCR expression was shown to promote tumor metastasis in lung adenocarcinoma^18^. Analysis of EPCR expression in lung adenocarcinoma tissues showed that EPCR expression correlates with clinical parameters^46^. EPCR is also shown to play a critical role in breast cancer growth in the orthotopic microenvironment of the mammary gland^19^. Our recent studies showed that EPCR provides an initial growth advantage to tumor cells in breast cancer^20^. In light of the above reports, it was totally surprising to find in our recent study that EPCR suppresses tumor growth in MPM^21^. Analysis of tumor tissue sections in this study revealed that tumors originated from MPM cell types expressing EPCR were prone to apoptosis.

At present, it is unclear how EPCR renders MPM cells susceptible to apoptosis. *In vitro* studies performed here show that EPCR expression, in itself, does not promote apoptosis in MPM cells. However, EPCR expression in MPM cells makes them highly susceptible to TNFα + IFNγ-induced cell death. It is unlikely that EPCR-APC or EPCR-FVIIa-mediated cell signaling is responsible for promoting TNFα + IFNγ-induced cell death of MPM cells since no APC or FVIIa was added in our experimental treatment. Furthermore, treatment of cells with EPCR blocking antibody that prevents APC and FVIIa binding to EPCR did not block the EPCR-mediated apoptosis (data not shown). Furthermore, all published literature using various other cell types showed that EPCR-APC-mediated cell signaling activates antiapoptotic and not proapoptotic pathways^3^,^4^,^49^. Consistent with this, we also found that addition of APC to MPM cells expressing EPCR reduced MPM cell apoptosis (data not shown). The proapoptotic function of EPCR appears to be limited to MPM cells as we found no significant differences in apoptosis in MDA231 breast cancer cells lacking noticeable EPCR levels and MDA 231 cells transduced to overexpress EPCR (data not shown).

Genome-wide expression profiling of mRNA in REN cells, REN cells transfected to express EPCR, MS-1, and M9K cells that constitutively express EPCR showed that EPCR expression alters the transcription profile in MPM cells. A most striking alteration is in the expression of cancer/testis (CT) antigens (GAGEs, XAGE 2B, MAGE, and CT45A4). Expression of these genes was markedly reduced, 50 to 100-fold, in MPM cells expressing EPCR (REN(+EPCR), MS-1, M9K) in comparison to REN MPM cells lacking EPCR. These data were confirmed in qRT-PCR (data not shown). In normal health, CT antigen expression is strictly restricted to the testes, but they are aberrantly expressed in various cancers^50^, including mesothelioma^51^. Recent studies suggest that CT antigens contribute to the pathogenesis of cancer by suppressing apoptosis and promoting cell survival^52^,^53^,^54^. GAGE was shown to render tumor cells resistant to apoptosis mediated by IFN-γ, Fas, taxol, and γ-irradiation^55^. Thus, it is possible that EPCR-mediated down-regulation of GAGE and other CT antigens in MPM cells makes EPCR expressing MPM cells highly susceptible to TNFα + IFNγ-induced apoptosis. A well-designed and in-depth study is needed to investigate this interesting hypothesis and to elucidate mechanisms by which EPCR suppresses the expression of CT antigens.

Consistent with our earlier report^21^, the data presented here show that EPCR exerts an antitumor effect *in vivo.* EPCR gene delivery to an established MPM xenograft in a mouse model significantly reduced the progression of tumor growth when compared to tumor growth observed in mice treated with a control vehicle or control adenovirus. It is interesting to note that administration of Ad.EPCR not only reduced tumor burden and volume, but it also decreased the total number of tumors in the thoracic cavity. This probably reflects a complete regression of some tumors, particularly small size tumors, following EPCR gene therapy. Minor antitumor effects seen with a control adenoviral vector might have come from the known activation of innate immune response by adenoviruses and the associated anti-tumor effects^56^. Analysis of EPCR protein expression in tumors of mice treated with Ad.EPCR by immunohistochemistry showed that EPCR protein expression in tumors was heterogeneous and limited to 15 to 25 cell layers deep. Cells in the tumor core appeared to be devoid of EPCR expression. This was expected as a replication-deficient adenoviral vector was used to deliver the EPCR gene, and it is unlikely that the adenoviral vector could penetrate into solid tumors beyond certain cell depth. This could also explain why Ad.EPCR treatment failed to completely prevent the progression of tumor growth.

It is interesting to note that EPCR transduction in tumor cells significantly increased the infiltration of macrophages and NK cells into the tumor microenvironment. At present, it is unknown how EPCR expression in MPM tumor cells attracts macrophages or NK cells into the tumor. EPCR-mediated cell signaling is known to elicit anti-inflammatory, and not proinflammatory, effects as it inhibits elaboration of proinflammatory cytokines by suppressing the activation of NFκB-induced gene expression^12^. It is possible that EPCR may function differently in MPM cells. Analysis of NFκB activation in MPM cells treated with TNFα + IFNγ showed no significant differences in the activation of NFκB in REN and REN(+EPCR) cells. Furthermore, measurement of IFNγ, TNFα, and IL1ß levels in the conditioned media of REN and REN(+EPCR) MPM cells cultured *in vitro* showed no significant differences between REN and REN(+EPCR) MPM cells (data not shown) unlike in the mouse thoracic fluids. It is possible that transduction of EPCR expression in tumor cells in combination with adenovirus vector-induced signaling and host inflammatory responses may be responsible for the marked increase in macrophage and NK cell infiltration into tumor microenvironment in mice treated with Ad.EPCR. The activation of innate immune response in the pleural space resulting in recruitment of macrophages and NK cells may be responsible for increased TNFα and IFNγ levels in the pleural space of mice treated with Ad.EPCR. Interestingly, in contrast to the increased levels of TNFα and IFNγ, the levels of other cytokines, both proinflammatory and anti-inflammatory, were significantly lower in the pleural lavage of mice treated with Ad.EPCR compared to mice treated with PBS vehicle or control adenovirus.

Consistent with our earlier observation that EPCR acts as a tumor suppressor in mesothelioma^21^, transduction of EPCR expression in MPM originating from MPM cells that are devoid of EPCR markedly increased apoptosis in tumors. The extent of tumor cell apoptosis appears more extensive than EPCR expression in tumor cells. This could be due to retention of apoptotic cells in the tumor. A marked increase in tumor cell apoptosis in mice administered with Ad.EPCR probably stems from the combined effect of EPCR-induced specific changes in the genome of MPM cells and increased levels of IFNγ and TNFα in the pleural space. Nude mice (NU/J) used in the present study lack T cells but their innate immunity is intact as they contain normal levels of macrophages, natural killer cells, and dendritic cells. Therefore, the antitumor effect of Ad.EPCR in the present study is attributable primarily to factors related to innate immunity and not to an acquired T-cell immunity. The present study does not provide any information on the specific contribution of various innate immune cell types to Ad.EPCR-mediated antitumor effect. However, based on the significant increase in infiltration of macrophages and NK cells into the tumor microenvironment in mice treated with Ad.EPCR, it is likely that macrophages and NK cells are the primary sources of the production of TNFα and IFNγ, respectively. Here, it may be pertinent to note that, although the principal source of IFNγ in the immune response is generally believed to be T cells (which are absent in NU/J mice used in the study) and NK cells, recent studies showed that activated macrophages were also capable of producing IFNγ upon appropriate cytokine stimulus^57^. Overall, the innate immunity appears to play a critical role in mediating Ad.EPCR-induced tumor cell apoptosis.

In the absence of standard medical therapies for MPM, and the localized nature of MPM and the relative ease in accessing the tumor in the pleural cavity, several groups investigated the potential therapeutic effect of intrapleural gene therapy in MPM patients in phase I clinical trials. These therapies include suicide gene therapy using suicide gene herpes simplex virus-1 thymidine kinase (HSV*tk*) and adenoviral vectors encoding interferon genes (see rev^38^). Although these studies showed safety, feasibility, and induction of anti-tumor humoral and cellular immune responses, their therapeutic effect is limited. Larger tumor volumes of MPM and immunosuppressive tumor microenvironments are some of the probable causes for limiting the efficacy of cytokine gene therapy. Preclinical models showed that inhibition of cycloxygenase-2 to mitigate the immunosuppressive tumor microenvironment by reducing PGE2 and IL-10 production^58^ or concomitant chemotherapy^59^ improved the efficacy of IFN gene therapy. In a recent pilot and feasibility clinical trial, the combination of intrapleural Ad.IFN, celecoxib, and chemotherapy proved to be safe in patients with MPM and significantly increased overall survival rate compared to historical controls^60^. The existing data, from both preclinical and clinical trials, indicate that intrapleural administration of Ad.IFN in MPM patients for immuno-gene therapy is safe, feasible, and promising, but combining the immuno-gene therapy with a second-line therapy improves the efficacy of the treatment^38^. EPCR gene therapy, if it does not meet the standard of being used as a primary therapeutic option, would at least improve the efficacy of immunotherapy. Several systemic immunotherapies have been administered to MPM patients, including the administration of IFNγ^61^,^62^,^63^,^64^,^65^. Although some positive responses have been seen, particularly in MPM patients with stage I disease^64^,^65^, overall the results were not as promising as it was hoped. It is possible that the poor response to IFNγ treatment in these patients could be due to the absence of EPCR expression in MPM cells that make them insensitive to IFNγ treatment. Combining EPCR gene therapy with IFN gene therapy may remove the necessity of using celecoxib and systemic chemotherapy to improve therapeutic efficiency^60^. The EPCR-mediated innate immune response would complement IFN-gene therapy induced T-cell-mediated immunity to elicit strong antitumor effects.

Overall, our present data suggest that intrapleural EPCR gene therapy has the potential to slow the progression of MPM but requires further refinement in the delivery and expression to make this option more viable. Our studies indicate that EPCR in MPM cells controls the progression of MPM by exerting its effect at multiple levels. First, EPCR in MPM cells elicits elaboration of cytokines TNFα and IFNγ *in vivo* and then renders MPM cells susceptible to TNFα + IFNγ-induced cell death. At present, it is unclear exactly how EPCR sensitizes the MPM cells to TNFα + IFNγ-induced cell death. Since the EPCR-mediated apoptotic effect in MPM cells appears to be independent of its known ligands APC and FVIIa, it is possible that the interaction of EPCR with yet to be unidentified endogenous ligand(s) unique to MPM cells may play a role in EPCR-mediated apoptosis in MPM.

## Material and Methods

### Cell lines

REN cells were from Steven M Albelda, University of Pennsylvania; MS-1 cells were from Su-Ming Hsu, The University of Texas Health Science Center at Houston, and M9K cells were from Brenda I Gerwin, National Cancer Institute. All three MPM cell types were obtained from the above investigators before 2008 by Steven Idell, The University of Texas Health Science at Tyler, who provided these cells to us as a part of our earlier collaborative studies^21^. Characterization of these cells, when they were first used in our MPM tumorigenesis studies, showed an epitheloid phenotype in culture and retained classic MPM markers, confirming their MPM origin^18^,^66^. All cells were grown in RPMI medium supplemented with 10% fetal bovine serum (FBS) and 1% penicillin/streptomycin (Gibco/BRL, Grand Island, NY) at 37 °C in a humidified 5% CO_2_ environment.

### Generation of stable transfectants of MPM cells expressing/lacking EPCR

REN cells that do not express EPCR were stably transfected with pZeoSV plasmid containing human EPCR cDNA^67^ to generate REN(+EPCR) cells^21^. EPCR was selectively knocked-down in MS-1 and M9K MPM cells that constitutively express EPCR by specific shRNA constructs cloned into pSilencer 2.1 U6-Puro expression vector. Details of the sequences of oligonucleotides used for the knock-down of EPCR gene and experimental procedures to generate MS-1(-EPCR), and M9K(-EPCR) were described in detail in an earlier publication^21^. The stable transfectants of MPM cells overexpressing EPCR or EPCR knocked-down were the same as that were used in our earlier study^21^. The efficiency of EPCR knockdown (>90% reduction in EPCR protein levels) and overexpression in the stable transfectants could be assessed from the data reported in the earlier publication^21^.

### Generation of adenoviral vectors encoding EPCR

Adenoviral vectors encoding either EPCR or GFP gene were constructed, expressed and amplified in HEK 293AD system. Briefly, HEK 293AD cells seeded in 60 mm dishes (80% confluent) were cotransfected with 1 μg of PacI-digested pacAd5 9.2–100 adenoviral backbone DNA and 5 μg of PacI-digested linearized pacAd5 CMVK-NpA shuttle vector containing EPCR cDNA using Fugene HD transfection reagent according to the manufacturer’s protocol (Roche Diagnostics Corp. Indianapolis, IN). After 7–8 days of transfection, HEK 293AD cells showing cytopathic effects were lysed by repeated freeze/thaw cycles and centrifuged at 3,000 × g to 10 min collect the supernatant containing primary adenoviral stock, which was used to infect HEK 293AD cells in 6–8 T-75 flasks to generate high titer viruses. Adenoviral particles were purified and concentrated using Fast trap adenovirus purification and concentration kit (Millipore Darmstadt, Germany). Viral titers were determined according to the manufacturer’s protocol using “Quick Titer Adenovirus Titer Immunoassay” kit (Cell Biolabs, Inc. San Diego, CA).

### Orthotopic murine model of thoracic human MPM

All experiments involving the use of animals in this study were approved by UTHSCT Institutional Animal Care and Use Committee and performed in accordance with the animal welfare guidelines outlined in the Guide for the Care and Use of Laboratory Animals. Details of orthotopic MPM murine model employed in the present study were described recently^21^. Briefly, MPM cells were detached from the culture dish using non-enzymatic cell dissociation reagent (MP Biomedicals, Solon, OH), washed once with phosphate buffered saline (PBS), and resuspended in sterile PBS containing growth factor-reduced Matrigel (BD Biosciences) at 1:4 dilution. One hundred μl of cell suspension containing 1 × 10^6^ cells were injected into the pleural cavity of Balb C nude (Nu/J) mice. Mice were sacrificed one month following tumor cell implantation; the chest cavities were photographed, and tumors were counted. Tumor volumes were calculated as described earlier^66^. All tumors measuring 2 mm or above were excised carefully from the thoracic cavity and weighed as an index for tumor burden. Tumor and tissue samples were fixed in Excell plus fixative (American Mastertech Scientific Inc.) overnight.

### EPCR gene therapy in murine MPM model

Nude mice were injected with 1 × 10^6^ REN MPM cells suspended in PBS containing growth factor-reduced Matrigel into the pleural cavity as described above. A group of mice was euthanized on the 11^th^ day to monitor the establishment and progression of the MPM tumors before starting EPCR adenoviral therapy. The remaining mice were randomly divided into three groups. One group received EPCR expressing adenovirus (Ad.EPCR), the second group received control (GFP-expressing) adenovirus (Ad.Control), and the third group received PBS. Adenoviral therapy started on the 11th day, and the animals received the adenoviral injections every third day for the next 20 days. Each mouse received a total of 2 × 10^9^ viral particles (in 100 μl of PBS) per injection into the pleural cavity. At the end of 30 days following the tumor cell inoculation, mice were euthanized, and tumor statistics were recorded. A total of three independent experiments were conducted.

### Pleural lavage

Before completely opening the chest cavity to measure tumor growth, pleural lavages were collected by pipetting 500 μl of sterile saline into the pleural space through a small opening made in the chest cavity. The ribcage was gently tapped, and the fluid was aspirated from the pleural cavity with the same pipette tip. The resulting fluid was centrifuged at 3,000 × g for 10 min to remove cells and cellular debris. The supernatants were stored at −80 °C until used.

### Immunohistochemistry

Fixed tissues were processed, sectioned and immunostained with anti-human EPCR IgG, macrophage marker F4/80, NK cell marker NK1.1 or TUNEL (for apoptosis) staining as described recently^21^. Briefly, tissues were processed using graded alcohol and xylene, embedded in paraffin, 5 μm-thin sections were cut and de-paraffinized using standard procedures. For immunostaining, rehydrated sections were processed for antigen retrieval using citrate buffer antigen retrieval protocol. Tissue peroxidases were inactivated by treating the tissue sections with 3% H_2_O_2_ for 30 min. Sections were then blocked with Dako antibody diluent solution and stained with control IgG, anti-human EPCR IgG, anti-F4/80 (Biolegend), NK1.1 (Biolegend), 5 μg/ml, or anti-BrdU (Promega, Madison, WI; 1:200 dilution) diluted in blocking buffer. Slides were washed to remove excessive primary antibodies, and tissue sections were labeled with biotinylated secondary antibodies followed by streptavidin-HRP using the Biotinylated Link Antibody Kit (Dako). Finally, sections were developed using AEC substrate chromogen and counterstained with hematoxylin followed by mounting with aqueous mounting media. Stained sections were viewed under a Nikon Eclipse Ti microscope and photographed using Nikon digital sight DS-Fi1 camera and NIS-Elements BR 3.2 software. The percentage of apoptotic cells was determined by counting the number of positively stained cells for anti-BrdU (TUNEL staining) among the total number of tumor cells counted in multiple randomly selected fields (20 to 25 fields) from 5 or more tumors obtained from different mice. A similar procedure was used to determine the percentage of macrophages and NK cells present in the tumor.

### Measurement of cytokines

Individual sandwich ELISA was used to measure the levels of TNFα, IFNγ, and IL-6 using commercially available kits (e-Bioscience, San Diego, CA). In addition, a panel of mouse cytokines and chemokines were measured in a magnetic bead-based multiplex assay employing Bio-Plex Pro Mouse Cytokine 23-plex Assay (Bio-Rad Laboratories) following the instructions provided with the product.

### Apoptosis assay/TUNEL staining

All apoptosis experiments were performed in complete medium. Briefly, 10^5^ cells/well were plated in a 6-well plate. Twenty-four hours after seeding the cells, the medium was replaced with fresh complete medium containing TNFα (10 ng/ml) and/or IFNγ (100 U/ml) for another 72 h. Cells were harvested using versene solution, and TUNEL staining was performed using DeadEnd Colorimetric TUNEL system (Promega) as per the manufacturer’s instructions and the extent of apoptosis was measured by flow cytometry.

### Statistical analysis

Nonparametric statistical tests were used to analyze the data. Analysis of variance (ANOVA) was used to analyze multi-group comparisons, and statistical significance levels were determined by non-parametric Kruskal-Wallis test, followed by Dunn’s multiple comparison post-test for determination of statistical significance between the two groups within the multi-group. Differences were considered statistically significant if *p* is <0.05. Statistical analysis was performed using GraphPad Prism v. 6 (GraphPad Software, San Diego, CA).

## Additional Information

**How to cite this article**: Keshava, S. *et al*. Intrapleural Adenoviral-mediated Endothelial Cell Protein C Receptor Gene Transfer Suppresses the Progression of Malignant Pleural Mesothelioma in a Mouse Model. *Sci. Rep.* **6**, 36829; doi: 10.1038/srep36829 (2016).

**Publisher’s note**: Springer Nature remains neutral with regard to jurisdictional claims in published maps and institutional affiliations.

## Figures and Tables

**Table 1:** Levels of various cytokines and chemokines in pleural lavage of mice implanted with REN MPM tumor cells at day 11 and then treated with PBS, control adenovirus, or EPCR adenovirus for 20 days as described in methods.

Cytokine/Chemokine	Before the treatment	PBS treatment	Control Adenovirus treatment	EPCR Adenovirus treatment
Eotaxin				
G-CSF	311.2 ± 20.84	635.4 ± 108.9^a^	367.3 ± 50.56^b^	389.1 ± 62.86
GM-CSF				
IL-1α	51.56 ± 8.918	43.49 ± 5.063	48.24 ± 9.109	31.54 ± 8.045
IL-1β	203.1 ± 74.25	110.0 ± 11.70	108.3 ± 21.48	164.0 ± 42.46
IL-2				
IL-3	151.0 ± 25.29	299.5 ± 52.80^a^	305.4 ± 69.44	139.5 ± 19.84^b,c^
IL-4	135.6 ± 33.85	344.3 ± 83.78^a^	261.8 ± 69.56	175.4 ± 37.95^b^
IL-5	70.17 ± 11.71	149.4 ± 29.21^a^	137.1 ± 31.34	136.0 ± 48.60
IL-6	71.85 ± 10.99	388.1 ± 81.22^a^	278.3 ± 71.84	196.8 ± 36.47^b^
IL-9				
IL-12 (p40)	811.0 ± 42.92	1635 ± 256.7^a^	1122 ± 135.0	1085 ± 206.4
IL-12 (p70)	152.95	758.4 ± 152.7	667.0 ± 162.8	370.4 ± 62.07^b^
IL-13	180.3 ± 35.73	268.3 ± 36.44^a^	197.0 ± 21.42	222.8 ± 21.89
IL-17A	712.8 ± 62.04	870.1 ± 78.61	1099 ± 145.0	1091 ± 115.5
KC	3121 ± 508.7	2042 ± 257.1	1343 ± 245.3	1272 ± 230.6^b^
MCP-1 (MCAF)	1336 ± 158.5	1912 ± 339.5	1154 ± 129.30^b^	1213 ± 109.9^b^
MIP-1α				
MIP-1β	154.8 ± 30.13	339.0 ± 33.89^a^	253.3 ± 38.98	255.6 ± 32.36^b^
RANTES	18.91 ± 8.020	45.69 ± 9.748^a^	56.70 ± 8.936	22.73 ± 4.781^b,c^

The values given are mean values ± SEM. Where no values were given, it indicates that they were either not detectable or out of the range in the bioplex ELISA assay. “a” indicates the value significantly differs (*P* < 0.05) from that of the value measured at the time of treatment began; “b” indicates that the value significantly differs (*P* < 0.05) from PBS treatment; “c” indicates that the value significantly differs (*P* < 0.05) from the control adenovirus. In the data set, a single outlier was identified using two-sided Dixon’s test for an outlier. This outlier was not included in the data analysis.
